# A Case of Nephrotic Syndrome With Minimal-Change Disease and Waldenstrom’s Macroglobulinemia

**DOI:** 10.4021/jocmr1387w

**Published:** 2013-10-12

**Authors:** Darren W. Grabe, Bo Li, Syed S. Haqqie

**Affiliations:** a Albany Medical College, 47 New Scotland Avenue, Albany, N.Y. 12208, USA; b Albany College of Pharmacy and Health Sciences, 106 New Scotland Avenue, Albany, N.Y. 12208, USA; c Albany Nephrology Pharmacy Group (ANephRx), USA; d St. Luke’s-Roosevelt Hospital Center, 1111 Amsterdam Ave, New York, NY 10025, USA

**Keywords:** Waldenstrom’s macroglobulinemia, Nephrotic syndrome, Minimal change disease

## Abstract

Kidney disease is a rare complication of Waldenstrom’s macroglobulinemia. We report a case of nephrotic syndrome and minimal change disease in a patient with biopsy proven Waldenstrom’s macroglobulinemia. The patient presented with over 12 grams of proteinuria and was successfully treated with oral prednisone over the course of 4 weeks. Repeat serum protein electrophoresis as well as serum immunoelectrophoresis revealed no paraproteins, urine analysis was negative for protein or blood by dipstick and spot urine protein was 9 mg/dL with creatinine of 101 mg/dL at time of last office visit. This case illustrates the successful treatment with corticosteroids alone with prolonged complete remission.

## Introduction

Kidney disease and kidney failure are rare complications of Waldenstrom’s macroglobulinemia (WM) [1, 2]. The kidney disease most often associated with WM is characterized by proteinuria and hematuria with the proteinuria rarely exceeding 2 grams in a 24 hour collection [3, 4]. Pathologic findings include IgM deposition in the glomerular basement membrane and infiltration by malignant B cells [4]. In one case, minimal change disease associated with WM was described with resolution chlorambucil and prednisone treatment [5]. Other notable cases include histopathological findings of mesangiocapillary glomerulonephritis in WM with resolution of nephrotic syndrome following treatment of cyclophosphamide, vincristine and prednisone [4]. We present a case of Waldenstrom’s Macroglobulinemia associated with nephrotic range proteinuria and biopsy-proven minimal change disease that responded to oral corticosteroids and remains in complete remission.

## Case Report

### Subjective

A 67-year-old, previously healthy, Caucasian man presented with a two week history of fatigue and weight gain of 30 pounds. Medication history included diclofenac 100 mg twice daily for more than 10 years. The patient did not smoke and drinking one to two alcoholic beverages a night. Review of systems was positive for decreased urine volume and occasional frothing. He denied dysuria, nocturia or hematuria.

### Physical exam

The patient presented fully alert and oriented to person, place and time with no discomfort. He weighed 103.2 kg, 5’11” inches, temperature 36.6 °C, blood pressure 104/88 mmHg and pulse 74 bpm. Back tenderness and some pain on rip hip flexion were appreciated. Extremities revealed 3+ pitting edema.

### Lab

Dipstick analysis of his urine revealed 4+ protein, 3+ blood and microscopic analysis of the sediment demonstrated numerous hyaline, broad, fatty and cellular casts. A 24-hour urinary protein collection showed 12.7 g protein including free kappa and lambda light chains and a serum protein electrophoresis showed an IgM Kappa restricted paraprotein. Serum albumin was 0.9 g/L and serum creatinine was stable at 1.0 mg/dL (eGFR = 78 mL/min/1.73m^2^) All other laboratory results were normal.

### Imaging

Kidneys were normal in size and morphology with no evidence of hydronephrosis or masses by ultrasound. Abdominal CT showed no evidence of lymphadenopathy, hepatosplenomegaly or organomegaly.

### Kidney biopsy

**Figure 1:**
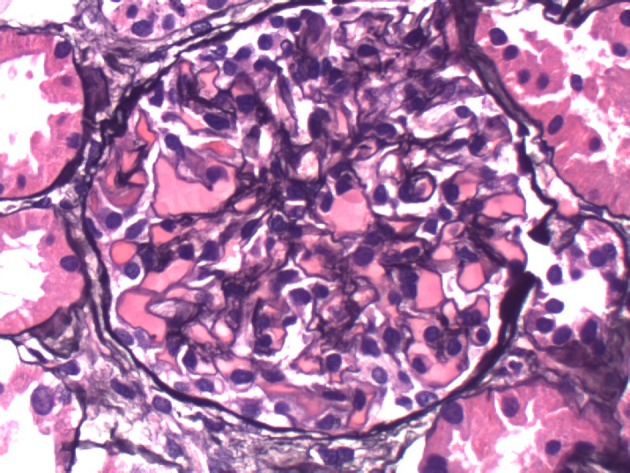
Basement membranes are not thickened. No double contours or basement membrane spikes are present. No segmental or nodular sclerotic lesions are present.

**Figure 2:**
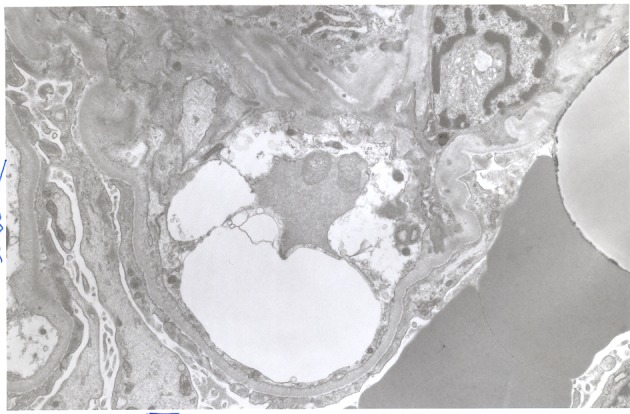
Electron micrograph demonstrating effacement of podocyte foot processes.

The kidney biopsy was obtained with light microscopy demonstrating minimal histological changes (Fig. 1). Electron microscopy showed evidence of focal global glomerulosclerosis (4/9 glomeruli were globally sclerosed), mild interstitial fibrosis, and tubular atrophy (Fig. 2). In the viable glomeruli, no significant increase in mesangial matrix or cellularity was observed. There were no double contours or basement membrane spikes observed. Capillary basement membranes were not thickened. There was also mild interstitial chronic inflammation, consisting of primarily lymphocytes and rare eosinophils. Renal tubules were back to back. Immunofluorescence showed focal, trace kappa with predominant uptake in tubular basement membrane. Ultrastructural evaluation of one glomerulus demonstrated diffuse epithelial foot process effacement over 90 percent. No evidence of amyloid, immune complexes, or cryoglobulin deposition was noted. Furosemide 20 mg twice daily was initiated however, he gained over 5.5 kg (weight 108.6 kg on admission) over two weeks following initial presentation and demonstrated pitting edema in his penis, scrotum, and both his lower extremities. The furosemide dose was increased to 40 mg twice daily with no demonstrable benefit for one week and the patient was subsequently admitted to further manage edema. Intravenous bumetanide (10 mg every 8 hours) and oral metolazone (5 mg twice a day) were initiated. The patient also received 2 exchanges of plasmapheresis followed by intravenous methylprednisone each time. Beta 2 microglobulin was 2.9 mg/L. Free kappa light chain was 134.33 mg/L. The free lambda light chain was 16.62 mg/L, and kappa/lambda ratio in the serum was 8.8 mg/L. Hepatitis A antibody, Hepatitis B surface antigen, hepatitis C antibody, ANA, Double-stranded DNA antibody were all negative, C3 and C4 were 147 and 25, respectively.

A bone marrow biopsy demonstrated a B-cell lymphoproliferative disorder with plasmacytic differentiation with overall cellularity of approximately 40% with a myeloid to erythroid ratio of 4:1. Paratrabecular and nodular interstitial lymphoid aggregates comprised of lymphocytes and plasma cells accounted for approximately 20% of cellularity. Plasma cells present were kappa dominant with kappa and lambda immunoglobulin light chain immunostains. Bone marrow aspirate study showed evidence for myeloid and erythroid marrow elements. Myeloid maturation and erythroid maturation was present. Blast forms were not increased in numbers, and megakaryocytes were not seen.

The patient was discharged with the diagnoses of nephrotic syndrome, minimal change disease with questionable Waldenstrom’s lymphoplasmacytic lymphoma. The patient was continued on prednisone 60 mg daily, furosemide 40 mg twice daily, and potassium chloride 40 mEq twice daily for 4 weeks. On follow-up 2 weeks after discharge, weight was 86.4 kg with 4.4 grams of protein in a 24 hour collection. The steroid taper was completed 6 months. Repeat serum protein electrophoresis as well as serum immunoelectrophoresis revealed no paraproteins, urine analysis was negative for protein or blood by dipstick and spot urine protein was 9 mg/dl with creatinine of 101 mg/dL at time of last office visit one year after initial presentation. Table 1 shows the weight and proteinuria for the patient over the course of his initial presentation to his follow-up visit 10 months later.

**Table 1:** Weight and Proteinuria for the Patient Over the Course

	Initial Presentation	Admission	Discharge	Follow Up 2 weeks later	Follow up 10 months later
Weight (kg)	103.2	108.6	94.6	86.4	94.1
24-hour urine (g)	12.7			4.4	
UA Protein	4+	3+		2+	Trace

## Discussion

WM is defined as the overproduction of IgM monoclonal proteins in the blood due to the infiltration of lymphoplasmacytic lymphoma in the bone marrow. WM is a rare disorder that affects three per million people per year with 1,400 new cases each year in the United States alone. It is most common in Caucasian males with a median age of 64 years at diagnosis. Etiology is unclear, however most recent theories include autoimmune reactivity to self antigens. Malignant B cells infiltrate hematopoietic tissues causing pancytopenia, lymphadeopathy, hepatomegaly and splenomegaly. Monoclonal IgM proteins produced by B cells can form pentameric configurations causing hyperviscosity syndrome. The monoclonal IgM produced can also act as an autoantibody to select against myelin-associated glycoprotein causing neuropathy or RBCs causing Coombs positive autoimmune cold hemolytic anemia. The monoclonal IgM protein can also deposit in extracellular spaces of the GI tract, skin or kidneys [6, 7]. Previous cases of nephrotic syndrome associated with WM were treated with cytotoxic agents and corticosteroids. This case illustrates the successful treatment with corticosteroids alone with prolonged complete remission.
